# The Evolution of Regulatory Elements in the Emerging Promoter-Variant Strains of HIV-1 Subtype C

**DOI:** 10.3389/fmicb.2021.779472

**Published:** 2021-11-16

**Authors:** Disha Bhange, Nityanand Prasad, Swati Singh, Harshit Kumar Prajapati, Shesh Prakash Maurya, Bindu Parachalil Gopalan, Sowmya Nadig, Devidas Chaturbhuj, Boobalan Jayaseelan, Thongadi Ramesh Dinesha, Syed Fazil Ahamed, Navneet Singh, Anangi Brahmaiah, Kavita Mehta, Yuvrajsinh Gohil, Pachamuthu Balakrishnan, Bimal Kumar Das, Mary Dias, Raman Gangakhedkar, Sanjay Mehendale, Ramesh S Paranjape, Shanmugam Saravanan, Anita Shet, Sunil Suhas Solomon, Madhuri Thakar, Udaykumar Ranga

**Affiliations:** ^1^HIV-AIDS Laboratory, Molecular Biology and Genetics Unit, Jawaharlal Nehru Centre for Advanced Scientific Research (JNCASR), Bengaluru, India; ^2^HIV Immunology Laboratory, Department of Microbiology, All India Institute of Medical Sciences (AIIMS), New Delhi, India; ^3^Division of Microbiology/Infectious Diseases Unit, St. John's National Academy of Health Sciences, Bengaluru, India; ^4^Department of Serology and Immunology, National AIDS Research Institute (NARI), Pune, India; ^5^Department of Molecular Biology and Genotyping, Y. R. Gaitonde Centre for AIDS Research and Education (YRG CARE), Chennai, India; ^6^Infectious Diseases Laboratory, Y. R. Gaitonde Centre for AIDS Research and Education (YRG CARE), Chennai, India; ^7^Department of Clinical Sciences, National AIDS Research Institute (NARI), Pune, India; ^8^Department of Research, P. G. Hinduja National Hospital and Medical Research Centre, Mumbai, India; ^9^YRGCARE Suniti Solomon Outpatient Clinic, Y. R. Gaitonde Center for AIDS Research and Education (YRG CARE), Chennai, India; ^10^Department of Medicine, School of Medicine, Johns Hopkins University, Baltimore, MD, United States

**Keywords:** HIV-1, subtype C, evolution, sequence duplication, latency

## Abstract

In a multicentric, observational, investigator-blinded, and longitudinal clinical study of 764 ART-naïve subjects, we identified nine different promoter variant strains of HIV-1 subtype C (HIV-1C) emerging in the Indian population, with some of these variants being reported for the first time. Unlike several previous studies, our work here focuses on the evolving viral regulatory elements, not the coding sequences. The emerging viral strains contain additional copies of the existing transcription factor binding sites (TFBS), including TCF-1α/LEF-1, RBEIII, AP-1, and NF-κB, created by sequence duplication. The additional TFBS are genetically diverse and may blur the distinction between the modulatory region of the promoter and the viral enhancer. In a follow-up analysis, we found trends, but no significant associations between any specific variant promoter and prognostic markers, probably because the emerging viral strains might not have established mono infections yet. Illumina sequencing of four clinical samples containing a coinfection indicated the domination of one strain over the other and establishing a stable ratio with the second strain at the follow-up time points. Since a single promoter regulates viral gene expression and constitutes the master regulatory circuit with Tat, the acquisition of additional and variant copies of the TFBS may significantly impact viral latency and latent reservoir characteristics. Further studies are urgently warranted to understand how the diverse TFBS profiles of the viral promoter may modulate the characteristics of the latent reservoir, especially following the initiation of antiretroviral therapy.

## Introduction

Based on phylogenetic association, the viral strains of HIV-1 are classified into four groups (M, N, O, and P), and within group M, into 10 different genetic subtypes, A, B, C, D, F, G, H, J, K, L (Yamaguchi et al., 2020), and numerous recombinant forms. Of the various genetic subtypes of HIV-1 unevenly distributed globally, HIV-1C and its recombinant forms are responsible for nearly half of the global infections (Locateli et al., 2007; Tebit and Arts, 2011; Hemelaar et al., 2019). Despite the high prevalence of HIV-1C, only a limited number of studies are available examining the causes underlying the expansion of these viral strains and their impact on disease manifestation.

Although the basic architecture of HIV-1 LTR is broadly conserved among the diverse HIV-1 genetic families, subtype-associated differences are manifested (Ramírez de Arellano et al., 2006). The configuration of transcription factor binding sites (TFBS), including those of NF-κB, NF-AT, AP-1, and other regulatory elements such as the TATA box, and the TAR region, in HIV-1C LTR (C-LTR) differs from that of the other viral subtypes (Ramírez de Arellano et al., 2006). Of the TFBS variations, differences in the copy number and sequence of the NF-κB motif are unique to HIV-1C. C-LTR typically contains three or four NF-κB motifs in the enhancer region compared to only one motif present in HIV-1A/E or two motifs in all the other HIV-1 subtypes (Bachu et al., 2012a). Further, the additional copies of the NF-κB motif in C-LTR are genetically variable, alluding to a possibility that the viral promoters are receptive to a diverse and broader range of cellular signals. For instance, the four copies of the NF-κB motif in the enhancer of 4-κB viral strains represent three genetically distinct NF-κB binding sites (Bachu et al., 2012a). Apart from the NF-κB motif, other regulatory elements, including AP-1, RBEIII, and TCF-1α/LEF-1, also show subtype-associated variations, although the impact of such variations has not been examined.

Several publications reported the insertion or deletion of TFBS in HIV-1 LTR. One example is the sequence duplication of the TCF-1α/LEF-1, RBF-2, AP-1, and c-EBPα binding motif in the modulatory region of the LTR, technically called the most frequent naturally occurring length polymorphism (MFNLP; Koken et al., 1992; Ait-Khaled et al., 1995; Estable et al., 1996; Zhang et al., 1997). Approximately 38% of the HIV-1B viral isolates contain MFNLP, a phenomenon believed to be a compensatory mechanism to ensure the presence of at least one functional RBEIII site in the LTR (Estable et al., 1998). Although RBEIII duplication has been found in several subtypes, the significance of this phenomenon has been examined predominantly in HIV-1B. It, however, remains inconclusive whether the presence of RBEIII duplication is directly associated with reduced viral replication or slower disease progression (Estable, 2007). A small number of reports reported RBEIII duplication in HIV-1C infection, however, without evaluating its effect on the replication fitness of the viral strains and disease progression (Rodriguez et al., 2007).

Over the past several years, our laboratory has documented the emergence of LTR variant strains of HIV-1C in India and elsewhere (Siddappa et al., 2004; Bachu et al., 2012b; Boullosa et al., 2014). While the appearance of genetic diversity and such diversity impacting viral evolution are common to the various genetic subtypes of HIV-1, the genetic variation we describe in HIV-1C is non-sporadic and radically different in an important aspect. Viral evolution in HIV-1C appears to be directional toward modulating transcriptional strength of the promoter by creating additional copies of the existing TFBS, such as NF-κB, AP-1, RBEIII, and TCF-1α/LEF-1 motifs, by sequence duplication and co-duplication. A single viral promoter in HIV-1 regulates two diametrically opposite functions critical for viral survival – transcriptional activation and silencing. Hence, any variation in the constitution of the TFBS (copy number difference and/or genetic variation) may have a profound impact on viral replication fitness.

Here, in a multicentric, observational, non-interventional, investigator-blinded, and longitudinal clinical study, we examined the promoter sequences of 455 primary viral isolates derived from ART-naïve subjects. We show that the magnitude of TFBS variation is much larger than we reported previously. At least nine different TFBS variant viral strains have emerged in recent years. Using the Illumina MiSeq platform, we attempted to characterize the proviral DNA of a selected subset of viral variants containing the RBEIII motif duplication. The data allude to the possibility that some of the emerging strains could achieve greater replication fitness levels and may establish expanding epidemics in the future, which requires monitoring. This work provides important insights into the HIV-1 evolution taking place at the level of population in India.

## Materials and Methods

### Study Participants and Samples

Participants were recruited at four different sites in India for primary screening (PS) and longitudinal study (LS) – All India Institute of Medical Sciences (AIIMS), New Delhi (PS=107, LS=73); National AIDS Research Institute (NARI), Pune (PS=61, LS=38); St. John’s National Academy of Health Sciences, Bangalore (PS=116, LS=60); and Y. R. Gaitonde Centre for AIDS Research and Education (YRG CARE), Chennai (PS=171, LS=37). Subjects above 18years of age with documented evidence of serological positive test for HIV-1 were recruited to the study. From the hospital records, all the study participants were reportedly ART-naive at baseline. Further, the approximate date of initial infection and the duration of the infection are not available for most of the participants. The study subjects were likely exposed to the viral infection for several years before they reported to the clinics.

### Ethics Statement

Written informed consent was obtained from all study participants, following specific institutional review board-approved protocols. Ethical approval for the study was granted by the Institutional Review Board of each clinical site. All the clinical sites screened the potential subjects, counselled, recruited the study participants, and maintained the clinical cohorts for the present study. The Human Ethics and Biosafety Committee of Jawaharlal Nehru Centre for Advanced Scientific Research (JNCASR), Bangalore, reviewed the proposal and approved the study.

### Primary Screening: LTR Amplification and Molecular Typing of the Viral Promoter

For the molecular typing of the viral promoter, 15ml of peripheral blood was collected from every participant at one time. Of this sample, 3–5ml of blood was allocated to determine the CD4 cell count by flow cytometry and the extraction of genomic DNA from whole blood. From the rest of the blood sample, PBMC were isolated by density-gradient centrifugation. The PBMC and plasma samples were stored in 1ml aliquots in a liquid nitrogen container or a deep freezer, respectively, for further clinical analysis.

Genomic DNA was extracted from 200μl of the whole blood using a commercial DNA extraction kit (GenElute™ Blood Genomic DNA kit, Cat. No. NA2020, Sigma-Aldrich, United States) and was eluted in 200μl volume. The extracted DNA samples from the clinical sites were shipped to JNCASR on cold packages. The LTR sequences were amplified using 200–300ng of genomic DNA as a template from each of the clinical samples using Taq DNA Polymerase (Cat. No. M073L, New England Biolabs, MA, United States) in a Peqstar 2x thermal cycler (Peqlab, VWR). The U3 region of LTR was amplified using a nested-PCR strategy with the primers listed in Supplementary Table S1. Strict procedural and physical safeguards were implemented to minimize carryover contamination that included reagent preparation and PCR setup, amplification, and post-PCR processing of samples in separate rooms. The PCR products were purified using a commercial DNA purification kit (FavorPrep Gel/PCR Purification Kit, Cat. No. FAGCK001, Favorgen Biotech Corp. Ping-Tung 908, Taiwan). The amplified LTR sequences were analyzed using Sanger Dideoxy sequencing (Applied Biosystems, CA, United States). All the sequences were subjected to further quality control by multiple sequence alignment and phylogenetic analysis with the in-house laboratory sequence database using the ClustalW algorithm of BioEdit sequence alignment editor and MEGA6.0 software, respectively. Different viral strains were categorized by analyzing the LTR spanning modulatory and enhancer region from the TCF-1α/LEF-1 motif up to the Sp1III motif.

### Phylogenetic Analysis

The phylogenetic analysis of the HIV-1C LTR variants derived from 455 patient samples was performed with 31 reference sequences representing different HIV-1 subtypes. The analysis was performed with 1,000 bootstrap values. The evolutionary history was inferred by using the maximum likelihood method based on the Tamura-Nei model (Tamura and Nei, 1993). The tree with the highest log likelihood (−36587.28) is shown. Initial tree(s) for the heuristic search were obtained by applying Neighbor-Join and BioNJ algorithms to a matrix of pairwise distances estimated using the maximum composite likelihood (MCL) approach and then selecting the topology with superior log likelihood value. The tree is drawn to scale, with branch lengths measured in the number of substitutions per site. The analysis involved 486 nucleotide sequences. A total of 292 positions were included in the final data set. Evolutionary analysis was conducted in MEGA7.0 (Kumar et al., 2016).

### The Follow-Up Clinical Procedures

Following successful characterization of the viral promoter at JNCASR, the clinical sites were advised to recruit specific study subjects without disclosing the nature of the viral LTR. The clinical sites were, thus, blinded to the identity of the viral LTR. All the clinical procedures were performed at the clinical sites using the same protocols and kits as described below.

A total of 15ml of peripheral blood were collected in a BD vacutainer (Cat. no. 367525, Becton Dickinson, CA, United States) from each participant at 0, 6, 12, 18, 24, and 36 months during 2015–19. The PBMC and plasma samples were stored in 1ml aliquots in a liquid nitrogen container or a deep freezer, respectively. The CD4 T-cell count was determined using the BD Multitest commercial kit-CD3/CD8/CD45/CD4 (Cat. No. 340491, Becton Dickinson, CA, United States) following the manufacturer’s instructions. The samples were analyzed using a BD FACSCalibur flow cytometer or any other suitable machine. Calibration of the flow cytometer was performed using BD CaliBRITE 3 and APC beads (Cat. no. 340486 and 340487, respectively, Becton Dickinson, CA, United States). The plasma viral RNA load was determined at 0 and 12-month time points using the Abbott m2000rt Real-Time PCR machine (Abbott Molecular Inc. Des Plaines, IL, United States). Levels of soluble CD14 (sCD14) in the plasma were determined using Human sCD14 Quantikine ELISA Kit (Cat No. DC140, R & D Systems, MN, United States). Analysis of sCD14 was performed at months 0 and 12.

### RNA Isolation and RT-PCR for the Next-Generation Sequencing

RNA was extracted from 1ml of the stored plasma samples using a commercial viral RNA isolation kit (NucliSENS miniMAG nucleic acid extraction kit, Ref. No. 200293, BioMerieux, France). The complementary DNA (cDNA) was synthesized using HIV-specific primers (Supplementary Table S1) and a commercial kit (SuperScript™ IV First-Strand Synthesis System with ezDNase™ Enzyme (Cat. No. 18091150, Invitrogen, Carlsbad, CA, United States). The reaction vials were incubated at 65°C for 5min, following 2-min incubation on ice and 50°C for 50min. The reactions were terminated by incubating the samples at 85°C for 5min, followed by RNaseH treatment. The cDNA was used for the amplification of LTR.

### The Next-Generation Sequencing

The PCR products containing the RBEIII motif duplication were subjected to the NGS analysis using the Miseq Illumina platform. Each sample was amplified in duplicates using primers containing a unique 8bp barcode sequence specific for each sample. The amplification of the U3 region (~300–350bp) using genomic DNA or cDNA prepared from plasma RNA was performed the same way as described above for the primary screening except that the primers contained a unique sequence barcode at the 5′-end as listed (Supplementary Tables S1, S2). The concentration of the purified PCR product was determined using the Qubit™ dsDNA BR assay kit (Cat. No. Q32850, Invitrogen, CA, United States). All the samples were pooled at an equal concentration and were processed further. We pulsed the LTR amplicon of Indie.C1, a reference HIV-1C molecular clone, as internal quality control for sequencing.

DNA was quantified using the QUBIT 3 Fluorometer and a dsDNA HS Dye. After adding an “A” nucleotide to the 3′ ends, the adenylated fragments were ligated with loop adapters and cleaved with the uracil-specific excision reagent (USER) enzyme. The DNA was further purified using AMPure beads and then enriched by PCR in 6cycles using NEBNext Ultra II Q5 master mix (Cat. No. E7645L, New England Biolabs, Inc., MA, United States), Illumina universal primer, and sample-specific octamer primers. The amplified products were cleaned by using AMPure beads, and the final DNA library was eluted in 15μl of 0.1X TE buffer. The volume of 1μl of the library was used to quantify by QUBIT 3 Fluorometer using dS DNA HS reagent. The analysis of the fragment size was performed on Agilent 4150 Tape Station by loading 1μl of the library to Agilent D1000 Screen Tape. The library was sequenced using the Illumina MiSeq system and MiSeq Reagent kit v3 (Cat. No. MS-102-3003, Illumina, San Diego, CA, United States) following the manufacturer’s instructions.

Data analysis was performed using a custom pipeline as depicted (Supplementary Figure S2). First, the quality assessment was performed using FastQC (version 0.11.5), and the sequencing adapters were removed using Trimmomatic-master (version 0.33) from the raw paired-end data. Second, the paired reads from both the forward and reverse files were merged using the PEAR algorithm of PANDAseq (version 3.9.1) with the minimum and maximum read length set to be 200 and 500, respectively, with an overlap of at least 8 base pairs. Next, the merged reads were mapped to the LTR region of the two reference sequences: Indie.C1 (AB023804.1) and D24 (EF469243.2), using local alignment in Bowtie2 software (version 2.3.5.1). Using custom C++ and shell scripts, the mapped reads were demultiplexed in individual samples based on the combination of forward and reverse barcodes (Supplementary Table S2). The reads containing the C-κB motif sequence (HIV-1C) were considered for further analysis using a custom C++ script. All the HIV-1C reads were then grouped in multiple categories based on the number of motifs and sequence of NF-κB, RBEIII, and TCF-1α/LEF-1 motifs using a custom C++ script. The percentage of every category was then calculated based on the total number of reads within each sample, using the custom shell, C++, and R scripts. Next, DeconSeq (version 0.4.3) was used to analyze and filter out inter-sample sporadic contamination. A reference database was prepared manually for every major-variant category (category with >10% of total reads at all time points), by taking only the most abundant variant of that category from each time point of the sample. The reference database of a variant category was used to cross-check for contamination in the same variant category of the other samples, where the variant category is <10% in one or more time points. The percent coverage and identity thresholds were set to 90% each to allow a maximum of 5 mismatches or indels in a sequence stretch of 50 bases. The sum of percentages of all the variants classified as clean for a category, in all the time points, was done using custom shell and R scripts. From the total number of reads for each time point of a sample, the number of contaminated reads was eliminated, and the clean reads were taken ahead for calculating the percentage prevalence of single and double-RBEIII variants.

### Statistical Analysis

The data were analyzed using GraphPad prism 9, except the sequences used for phylogeny determination. *p* values of 0.05 or less were considered statistically significant. A non-parametric, Kruskal-Wallis (for multiple comparisons) test was applied to evaluate statistical significance in the case of a cross-sectional analysis of plasma viral load. One-way ANOVA was used to evaluate statistical significance for cross-sectional analysis of CD4 cell count and sCD14 levels. Two-way ANOVA was used to evaluate statistical significance in the case of longitudinal analysis of all the three parameters, plasma viral load, CD4 cell count, and sCD14.

### Data Availability Statement

All the sequences reported in this paper are available from the GenBank database under accession nos. MN840242.1–MN840356.1, MT847032–MT847207, and MT593868–MT594037. The raw data files for Illumina MiSeq are available under accession no. PRJNA720640.

## Results

### The Magnitude of TFBS Variation in HIV-1C LTR

**Figure 1:**
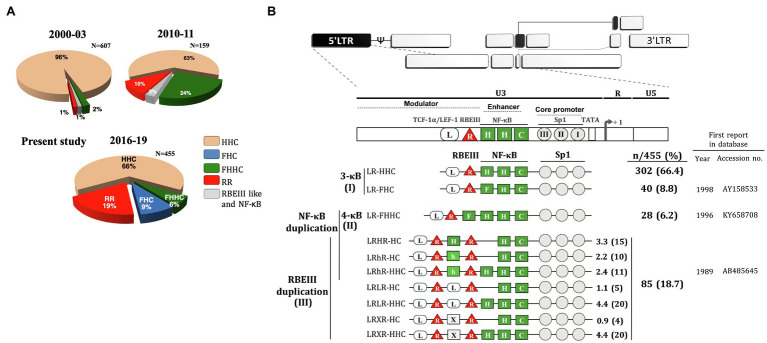
Profile of HIV-1C promoter variants in the Indian population. **(A)** The time of sample collection, sample number, and the nature of the promoter configuration are illustrated. The pie charts represent the percentage prevalence of the variant viral strains as color-coded – canonical HHC (beige), FHC (blue), FHHC (green), RR (duplication of RBEIII; red), and duplication of RBEIII like and NF-κB (grey). The data for the periods 2000–2003 and 2010–2011 are adapted from Bachu et al. (2012a) and replotted. The data of the present study are presented in the pie chart 2016–19. **(B)** The magnitude of TFBS variation in HIV-1C LTR. The upper panel represents the genome organization of HIV-1 followed by the TFBS arrangement in the canonical HIV-1C LTR. Sp1 motifs are depicted as grey circles, RBEIII motifs (R) as red triangles, and TCF-1α/LEF-1 sites (L) as open rectangular boxes. The various types of NF-κB binding sites (H, C, F, and h) are depicted as green square boxes. The various HIV-1C viral strains are classified into three main categories based on the NF-κB and/or RBEIII motif duplication. (I) The 3-κB LTR viral strains. (II) The canonical 4-κB LTR viral strains. (III) The viral strains containing the RBEIII site duplication. The two RBEIII sites are separated by an interceding sequence that constitutes an additional copy of a κB-motif (H), κB-like motif (h), TCF-1α/LEF-1 motif (L), or sequence without a distinct pattern (X). The analysis represents 455 of the 518 LTR sequences, and we could not type 63 other sequences.

**Figure 2:**
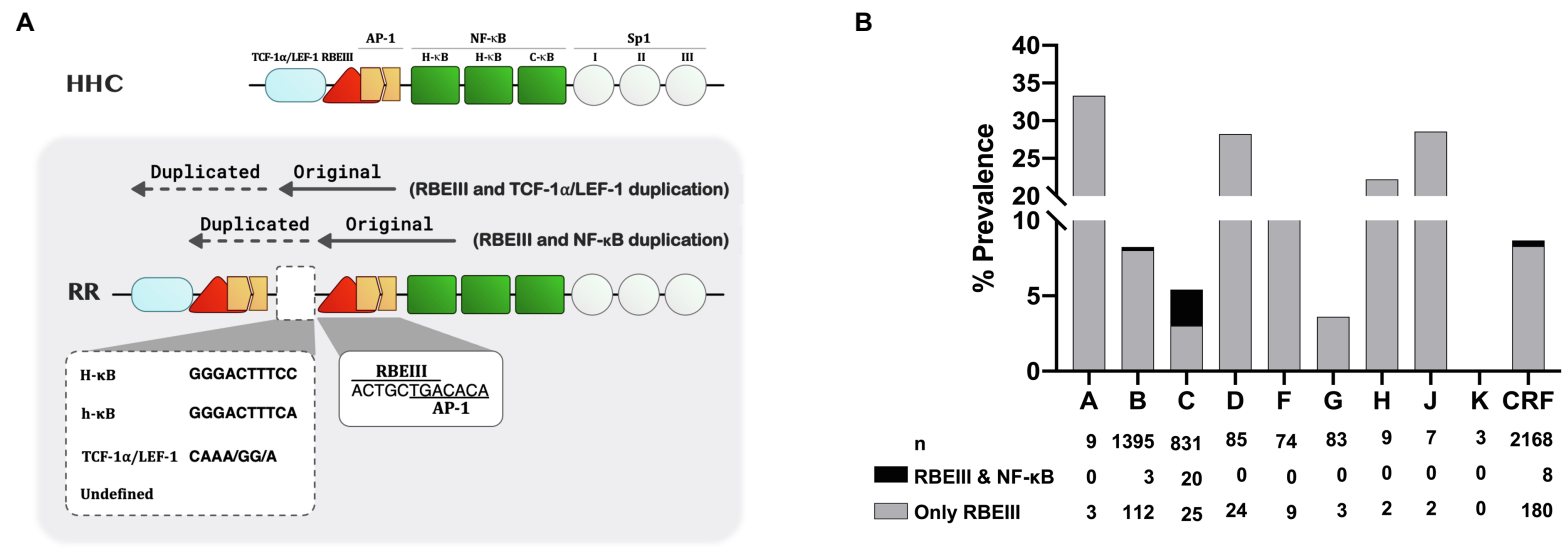
The nature of the RBEIII cluster duplication in HIV-1C. **(A)** A schematic representation of the RBEIII cluster duplication in the RR group of HIV-1C variants. The top panel depicts the arrangement of TFBS in the canonical HHC-LTR. The bottom panel portrays the RBEIII cluster duplication (RR, two RBEIII clusters) in two variant LTRs – the co-duplication of RBEIII and TCF-1α/LEF motifs or RBEIII and NF-κB motifs. Of note, the RBEIII cluster duplication comprises the copying of a sequence that recruits both RBF-2 and AP-1 (c-Jun and ATF) collectively. The original and duplicated sequences are marked with solid and dotted arrows, respectively. The two RBEIII clusters are typically separated from each other by an intervening sequence that constitutes a binding site for NF-κB (a canonical H-κB site or a non-canonical h-κB site), TCF-1α/LEF-1 motif, or a sequence of undefined character. **(B)** The relative prevalence of the RBEIII and/or NF-κB motif duplication in HIV-1 subtypes. All the available LTR sequences containing the duplication of one or both TFBS were downloaded from the LANL HIV database and categorized under the major HIV-1 subtypes as shown. A single sequence per patient was included in the analysis. The grey and black bars represent the prevalence of duplication of RBEIII and NF-κB motifs, respectively. n: the number of sequences, CRF: Circulating recombinant forms.

We collected 764 primary clinical samples from four different clinical sites in India, All India Institute of Medical Sciences (AIIMS), New Delhi; National AIDS Research Institute (NARI), Pune; St. John’s National Academy of Health Sciences, Bangalore; and Y. R. Gaitonde Centre for AIDS Research and Education (YRG CARE), Chennai, between 2016 and 2019. Using the genomic DNA, we could successfully amplify the U3 region in the LTR of 518 of 764 viral samples, whereas the amplification of the rest of the samples failed. The sequences of the 518 amplified PCR products were determined by the Sanger sequencing method. Of the 518 sequences, the genetic typing of only 455 could be accomplished. The sequence analysis indicated a rapidly changing TFBS profile in HIV-1C (Figure 1A). The proportion of viral variants across the four clinical sites was comparable without geographic skewing (Table 1). A large majority of the variant LTRs contain additional copies of the existing TFBS, including that of NF-κB, RBF-2, AP-1, and TCF-1α/LEF-1 binding sites (Figures 1B, 2A). Based on the TFBS profile, the sequences of the copied TFBS, and their temporal location, the various viral promoters may be classified into three categories.

**Table 1:** Proportion of promoter variants at each clinical site.

Category	Variant	AIIMS	NARI	St. John’s Hospital	YRG CARE	All clinics
I	HHC	66 (61.6%)	38 (62.3%)	79 (68.1%)	119 (69.6%)	302 (66.4%)
	FHC	13 (12.2%)	5 (8.2%)	8 (6.9%)	14 (8.2%)	40 (8.8%)
II	FHHC	8 (7.5%)	6 (9.8%)	9 (7.8%)	5 (2.9%)	28 (6.2%)
III	RR	20 (18.7%)	12 (19.7%)	20 (17.2%)	33 (19.3%)	85 (18.7%)
Total		107	61	116	171	455

Category-1 is represented by the viral promoters containing three NF-κB motifs without the duplication of any other TFBS. This group consists of the canonical LR-HHC-LTR and a new variant LR-FHC-LTR. The canonical LR-HHC-LTR represents the largest group among all HIV-1C promoters, comprising 302 of 455 sequences (66.4%). This LTR contains three tandemly arranged NF-κB sites in the enhancer, representing two distinct κB-motifs, two H-κB motifs (5′-GGGACTTTCC-3′), and one C-κB motif (5′-GGGGCGTTCC-3′, differences underlined). Immediately upstream of the viral enhancer, an RBF-2 binding site (R, RBEIII motif, 5′-ACTGCTGA-3′) and further upstream a TCF-1α/LEF-1 site (L, 5′-TACAAA/GG/A-3′) are located. The canonical HIV-1C promoter is identified here as LR-HHC-LTR to denote the 5′ to 3′ arrangement of the three kinds of TFBS, TCF-1α/LEF-1, RBEIII, and NF-κB. The second member of category-1 contains a variant LR-FHC-LTR comprising 40 of 455 (8.8%) sequences. The characteristic feature of these viral strains is the presence of three genetically distinct NF-κB motifs in the viral enhancer. Thus, the two viral strains of category-1 formed the major proportion of all the LTRs, 342/455 (75.2%; Figure 1). Multisequence alignments of several representative viral strains of the canonical LR-HHC (Supplementary Figure S1A) and the variant LR-FHC (Supplementary Figure S1B) are presented. The LR-FHC-LTR, being reported here for the first time, may have originated from the 4-κB viral strain FHHC of category 2, as described below.

Category-2 viral LTRs contain an additional (fourth) NF-κB binding site (F-κB site, 5′-GGGACTTTCT-3′) located downstream of the RBEIII site. The 4-κB LTRs, thus, contain one F-, two H-, and one C-κB motifs, in that order, hence are labeled LR-FHHC. An alignment of several representative 4-κB viral sequences shows a high degree of sequence conservation at all the TFBS (Supplementary Figure S1C).

### Double-RBEIII LTRs Show Profound Variation in Number, Genetic Sequence, and Position of TFBS

Category-3 viral strains, representing 18.7% (85/455) sequences analyzed here, are characterized by the duplication of the RBEIII motif (Figure 1B), a duplication analogous of MFNLP described previously in HIV-1B (Estable et al., 1996). This group is denoted as “RR” to signify the duplication of the RBEIII motif (R; Figure 2A). Of note, although the RBEIII motif duplication is common to all the HIV-1 subtypes (Estable et al., 1998; Gómez-Román et al., 2000), a co-duplication of RBEIII and NF-κB motifs is unique to HIV-1C, not seen in other HIV-1 subtypes (Figure 2B). Several unique molecular properties qualify the duplication of the RBEIII site, as described below.

Firstly, the duplicated sequence consists of three different elements – two of the elements invariably coduplicated, along with a third variable element. The two elements coduplicated are the eight base RBEIII core motif (5′-ACTGCTGA-3′) which binds the RBF-2 factor, and a downstream seven base motif (5′-TGACACA-3′) that forms a binding site for AP-1 factors (Jun, Fos, or ATF; Figure 2A). Notably, the RBEIII core sequence overlaps with the binding site of the AP-1 factor. Thus, a part of the duplicated sequence (5′-ACTGCTGACACA-3′,) appears to mediate the binding of a TF complex consisting of RBF-2, AP-1, and this cluster is highly conserved in the double-RBEIII LTRs or RR group. Secondly, in addition to the duplication of the 5′-ACTGCTGACACA-3′ sequence, additional sequences are also coduplicated, forming the basis for further classification of the viral strains. In a canonical HIV-1C LTR, the RBEIII motif is flanked by an upstream TCF-1α/LEF-1 site (5′-TACAAA/GG/A-3′) and a downstream NF-κB element (5′-GGGACTTTCC-3′). When the RBEIII motif is duplicated, one of these two TFBS is also coduplicated serving as an intervening sequence, forming at least two subgroups – viral strains containing the co-duplication of a TCF-1α/LEF-1 or NF-κB site. Thirdly, the variant LTRs contain three or two NF-κB motifs in the enhancer region.

Based on the nature of the intervening sequence, the viral strains may be classified into two major subgroups, one containing the presence of a canonical NF-κB motif (5′-GGGACTTTCC-3′, LRHR-HC), or κB-like motif (5′-GGGACTTTCA-3′, which is denoted as “h” κB-motif in the present work, strains LRhR-HC and LRhR-HHC) and the second subgroup with a TCF-1α/LEF-1 (5′-TACAAA/GG/A-3′, LRLR-HC, and LRLR-HHC) or an incomplete TCF-1α/LEF-1 binding sequence. A third subgroup may also be identified where the intervening sequence does not seem to form a binding site for a defined host factor (LRXR-HC and LRXR-HHC). Multisequence alignments of the variant viral promoters are presented (Supplementary Figures S1D–J). Of note, the three NF-κB motifs in LRHR-HC, LRhR-HC, and LRhR-HHC strains are not arranged in tandem, thus, blurring the distinction between the viral enhancer (consisting of the only NF-κB motifs) and the upstream modulatory region.

**Figure 3:**
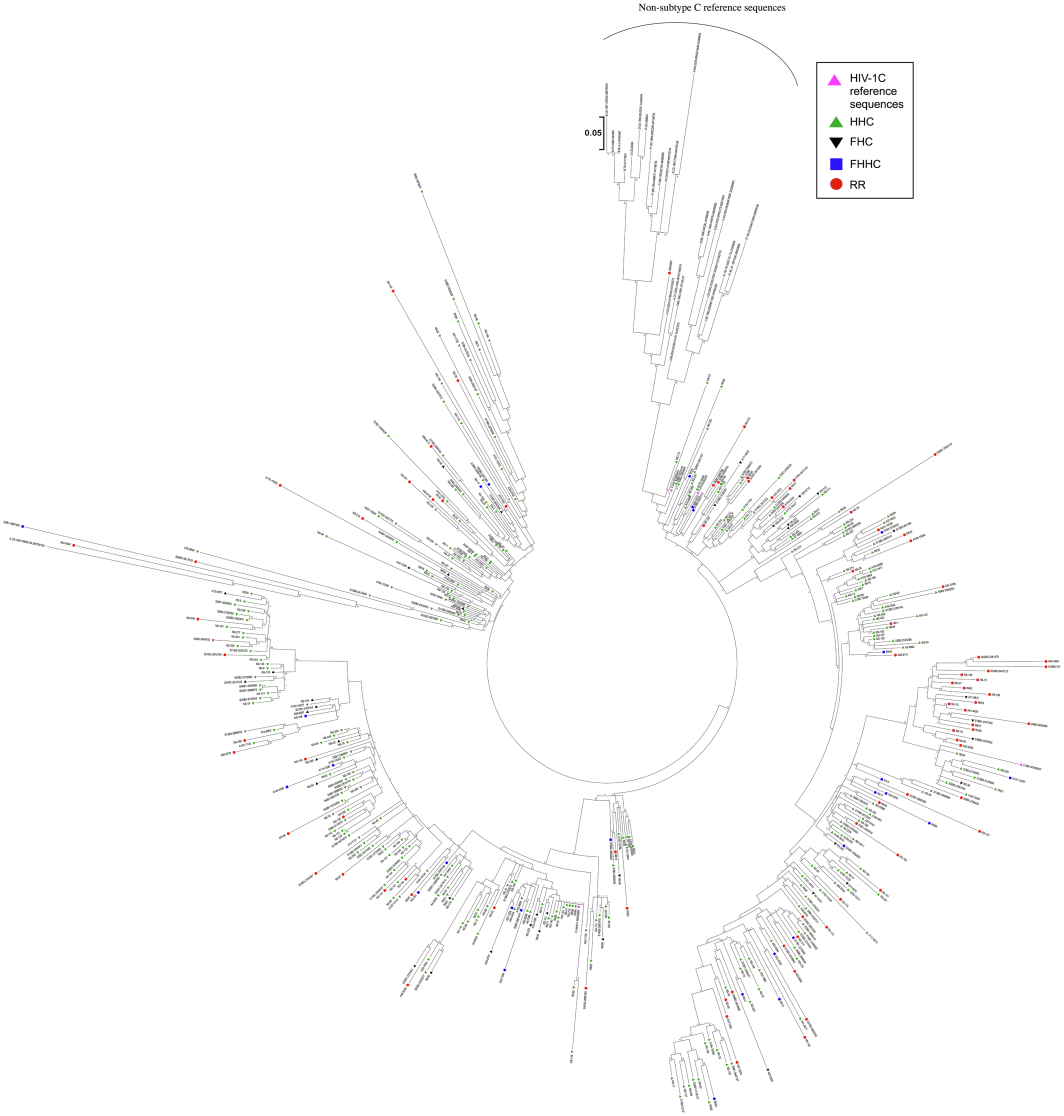
Phylogenetic analysis of HIV-1C LTR variant sequences. A total of 455 viral sequences isolated from study participants are included in the analysis. The analysis also includes four HIV-1C reference sequences and three sequences representing each primary genetic subtype of HIV-1, as described in materials and methods. Different LTR variants are represented using different symbols and colors, as depicted. The evolutionary history was inferred by using the maximum likelihood method based on the Tamura-Nei model. The analysis was performed with 1,000 bootstrap values. The tree is drawn to scale, with branch lengths measured in the number of substitutions per site. There were a total of 292 positions in the final data set. Evolutionary analyses were performed using MEGA 7.0 software.

In a phylogenetic analysis of 455 LTR sequences, compared with 31 reference sequences, all the viral strains of the cohort grouped with HIV-1C ascertaining their genetic identity (Figure 3). Notably, the various promoter variant viral sequences combined homogeneously, without forming separate clusters based on the TFBS variation.

### Longitudinal Analysis of Prognostic Markers

Increased transcriptional strength of the LTR may augment plasma viral load modulating various prognostic markers and immune activation markers. To this end, we monitored a few prognostic markers, such as the plasma viral load, CD4 cell count, and soluble CD14 levels, in the blood samples at the baseline and at two or three follow-up time points spaced 6months apart. Unfortunately, this objective could be fulfilled only partially given practical constraints. Following the primary screening and typing of the viral promoters, we could recruit only 208 of the 455 study participants for the follow-up analysis. Subsequently, with the implementation of the “Test and Treat” policy in 2017 in India, several participants enrolled for the follow-up analysis were excluded from the study. Consequently, the longitudinal analysis could be accomplished only with a small number of study participants, who did not prefer to switch to ART.

The demographic features of the 208 study participants at the baseline are summarized (Table 2). Of the 208 study participants, the percentage of female, male, and transgender are 55.3% (115/208), 43.8% (91/208), and 1.0% (2/208), respectively. The average (mean) age of the study participants was 34.4±8.45years (median=33years). For the subsequent analyses, all the viral strains were classified into four categories based on the nature of the TFBS variations identified in the viral promoter – HHC (the conventional LTRs containing the HHC κB-binding sites), FHC (all the three κB-binding sites are genetically distinct in these LTRs), FHHC (the LTRs contain four κB-binding sites), and RR (double-RBEIII strains; the seven viral variant strains are pooled into a single category, given the limited number of samples in individual groups).

**Table 2:** Characteristics of study participants at the time of recruitment.

Parameters	
Total number of participants	208
**Gender**	
Female	55.3% (*n* =115)
Male	43.8% (*n* =91)
Transgender	1.0% (*n* =2)
**Age (years)**	
Mean and SD	34.4 ± 8.45
Median	33
ART status	Naïve

**Figure 4:**
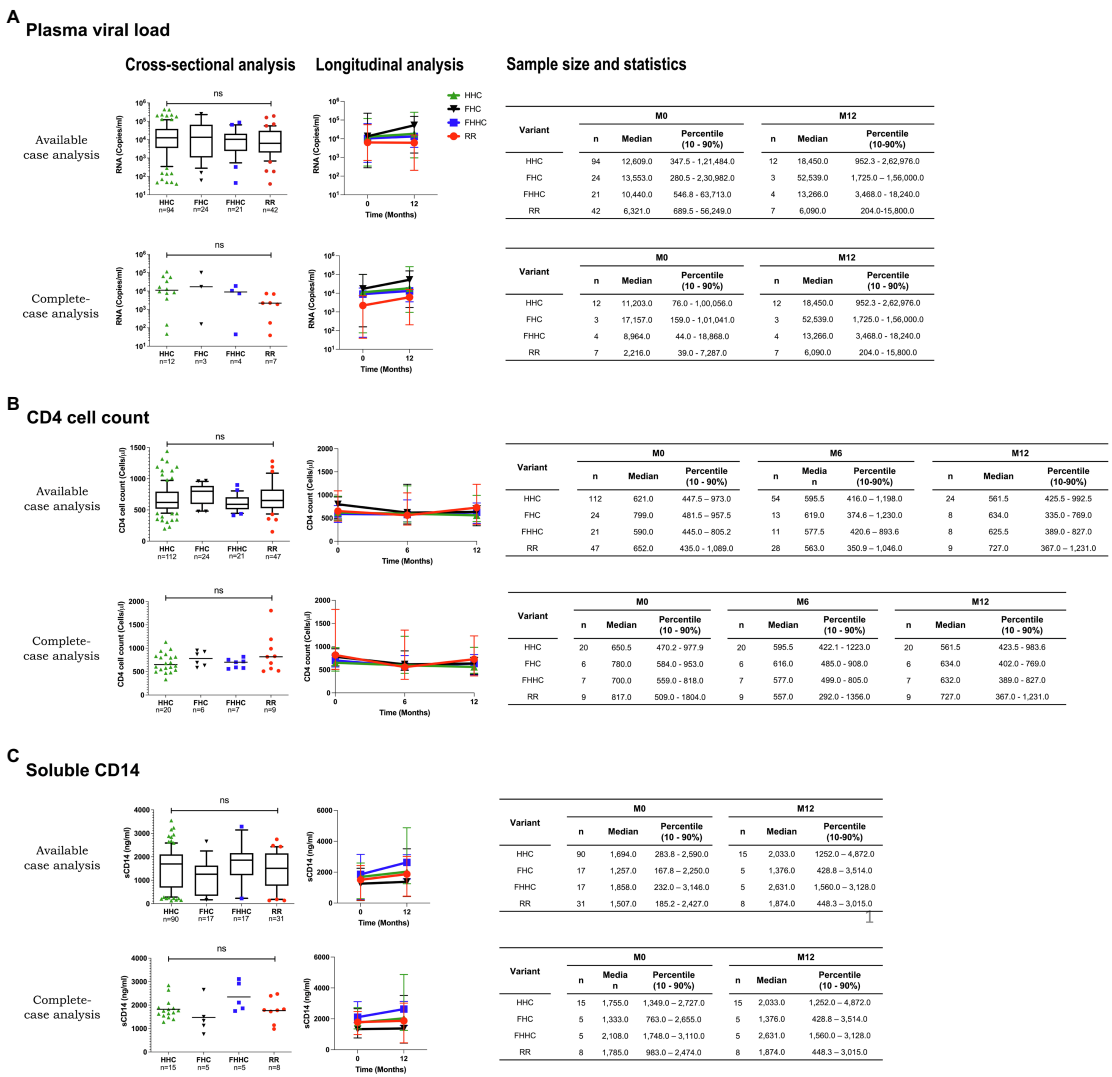
Cross-sectional and longitudinal analyses of prognostic markers. **(A)** Plasma viral load, **(B)** CD4 cell count, and **(C)** soluble CD14 levels of the four groups are presented at the baseline (left panels) and follow-up points (right panels). An available-case and complete-case analysis is presented for all the prognostic markers. The sample size used in each evaluation and the corresponding statistics are presented, in the tables. Given the limited sample numbers, the seven RR groups were pooled into a single double-RBEIII arm. Different LTR variant types are represented using different symbols and colors, as depicted. A non-parametric test, that is, the Kruskal-Wallis test, was applied for the statistical analysis of the plasma viral load. One-way ANOVA with Dunnett multiple comparison test was applied to CD4 cell count and sCD14. Two-way ANOVA was used for the comparison of the longitudinal analysis.

We compared the levels of plasma viral load, CD4 cell count, and sCD14 among these four categories at the baseline in a cross-sectional analysis. This analysis did not show a significant difference in any of the three parameters among the four groups (Figure 4, left panels). At M0, the median PVL values for the HHC, FHC, FHHC, and RR groups were 12,609.0, 13,553.0, 10,440.0, and 6,321.0 copies/ml, respectively (Figure 4A, left panel). The median values of CD4 cell count and sCD14 were also similarly comparable among the four groups. We also compared the three clinical parameters between the baseline and 12M time point for PVL and sCD14 and baseline, 6M and 12M for CD4 cell count (Figure 4, right panels). All the three parameters appeared to remain stable without a significant change between the baseline and 12M. The promoter configuration did not appear to make a significant difference for any of the three parameters examined. For instance, the median PVL values at 12M were 18,450.0, 52,539.0, 13,266.0, and 6,090.0 copies/ml, for HHC, FHC, FHHC, and RR groups, respectively. The CD4 cell count remained stable over the 12-month observation period among all four groups. The sCD14 levels appeared to show an increasing trend among all four groups at the follow-up; these differences, however, were not statistically significant. Of note, in the complete-case analysis, a trend of low-level plasma viral load was manifested for the RR variants compared to the three other groups (Figure 4A, right panel). However, the viral load increased between the time points among all the groups.

### The Coexistence of Viral Variants in Natural Infection

Of the various promoter variant viral strains described in the present work, the emergence of LTRs containing RBEIII duplication is relevant to HIV-1 latent reservoirs. In the context of HIV-1B, the RBEIII site and the AP-1 motif are known to play a predominantly suppressive role, especially in the absence of cellular activation (Naghavi et al., 1999; Bernhard et al., 2013; Kropp et al., 2014). The relative proportion of reads representing single vs. double-RBEIII motif-containing viral sequences in a coinfection may offer leads as per the biological significance of RBEIII duplication in natural infection.

To this end, we identified a subset of four of 85 subjects of our cohort who showed the presence of a coinfection of single- and double-RBEIII viral strains in Sanger sequencing. The clinical profile of the four subjects (2079, 3767, 4084, and VFSJ020) is summarized (Table 3). We performed an NGS analysis, using the MiSeq Illumina platform (Supplementary Figure S2), of the whole blood genomic DNA and plasma viral RNA of the four subjects at the baseline and two or three follow-up time points.

**Table 3:** The clinical profile of the four study subjects containing a duplication of the RBEIII motif.

Subject ID	Age/ Gender	Enrollment date	Promoter variant	PVL (number of RNA copies/ml)		CD4 (cells/μl)			sCDS14	
				M0	M12	M0	M6	M12	M0	M12
2079	32/F	01/06/2016	LR-HHC and LRLR-HHC	185	9,989	989	332	558	1,146.0	729.6
3767	40/F	29/06/2016	LR-HHC and LRhR-HHC	3,742	-	566	292	661	139.0	-
4084	38/F	07/05/2016	LR-HHC, LR-HC, and LRhR-HC	6,924	5,179	509	415	442	983.0	448.3
VFSJ020	38/F	22/09/2016	LR-HHC and LRhR-HC	21,200	-	461	-	579	2,204.4	-

**Figure 5:**
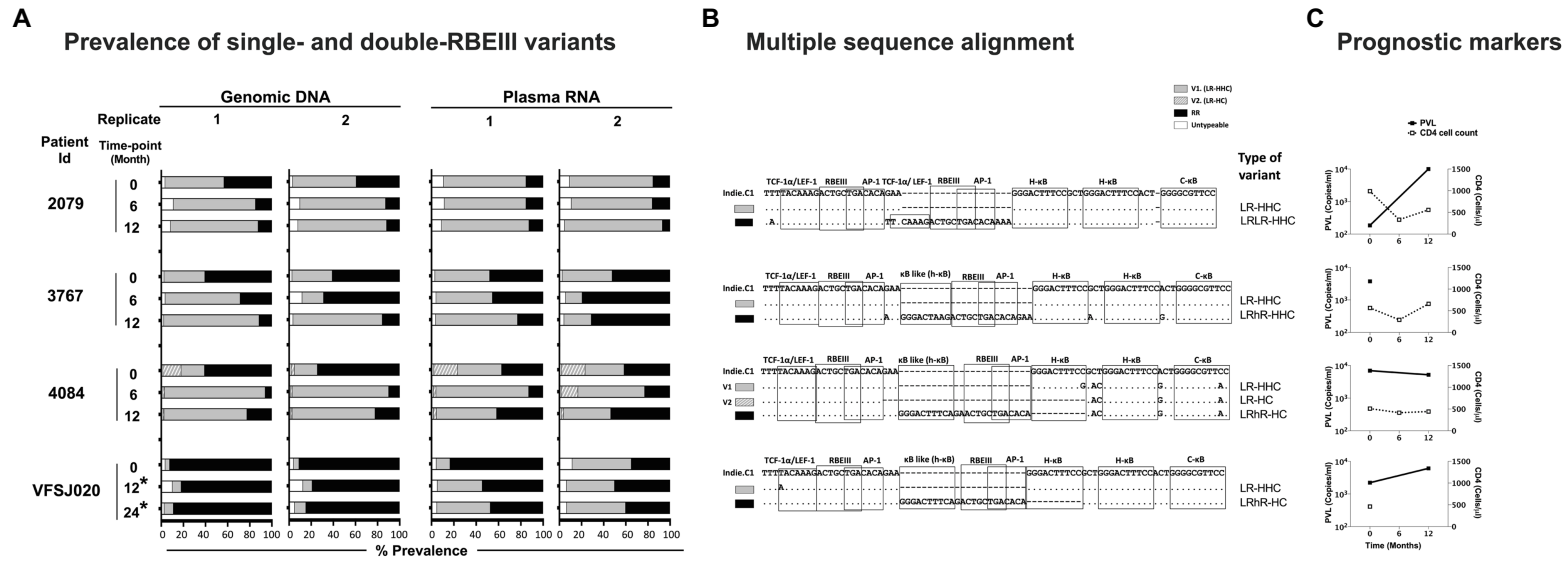
The frequencies of single- and double-RBEIII variants in a subset of study participants. **(A)** Two independent analyses were performed (replicates 1 and 2) using both the whole blood genomic DNA and plasma viral RNA. The samples were collected at six-month intervals, as shown. An asterisk (*) represents the samples collected post-ART. The dark, grey, and hollow bars represent the percentage prevalence of double-RBEIII, single-RBEIII, and minority/un-typable viral strains, respectively. **(B)** Multiple sequence alignment of single- and double-RBEIII promoter variants in respective subjects, as indicated. TFBS of relevance are marked using open square boxes. The viral variants are aligned with the Indie.C1 reference sequence, which was pulsed in the sequencing sample as an internal control. Dashes and dots represent sequence deletion and sequence homology, respectively. **(C)** Prognostic markers, plasma viral load (PVL), and CD4 cell count are represented by a filled box with a solid line and an open box with a dotted line, respectively.

The NGS data confirmed the coexistence of single- and double-RBEIII viral strains in all four subjects in both the DNA and RNA compartments. Importantly, in two subjects (2079 and 4084), the single-RBEIII strains represented a significantly larger proportion of reads in the plasma RNA compared to the double-RBEIII strains (Figure 5). Only in subject VFSJ020, the double-RBEIII reads dominated the single-RBEIII reads at all the time points, whereas in subject 3767, a mixed profile was observed. The data between the replicate samples are consistent with each other ascertaining the reproducibility of the analysis. A broad-level concurrence between the plasma RNA and genomic DNA was also noted.

## Discussion

The key finding of the present work is the continuing evolution of the HIV-1C viral promoter and as a consequence, the emergence of new variants at the population level. In 2004, we reported the emergence of HIV-1C strains containing four copies of the NF-κB binding motif in the viral enhancer for the first time in India (Siddappa et al., 2004; Bachu et al., 2012b). The 4-κB viral strains dominated the canonical viral strains containing three copies of NF-κB motifs in natural infection and under all experimental conditions, alluding to the additional copy of NF-κB motif conferring replication advantage (Bachu et al., 2012a). Subsequently, the 4-κB viral strains were also detected in Brazil and several African countries, suggesting that the phenomenon is not specific to a single country (Boullosa et al., 2014). Although our initial focus was limited to the NF-κB motif duplication and its impact on viral gene expression, we also observed the duplication of other TF binding motifs, including RBEIII and AP-1, though at a lower frequency (Bachu et al., 2012a,b).

### Sequence Motif Duplication in HIV-1C Differs From That of Other HIV-1 Families

Gene duplication accompanied by sequence variation played a crucial role in the acquisition of novel properties leading to the evolutionary success of organisms (Carroll, 2005). In viruses, the duplication of biologically important sequence motifs may confer the same survival advantage as gene duplication has done in higher organisms (Kropp et al., 2014). The significance of sequence motif duplication in the coding sequences of HIV-1 has attracted more attention conventionally compared to that of regulatory sequences (Marlowe et al., 2004; Guglietta et al., 2010; Sharma et al., 2018). Further, numerous publications reported the deletion or duplication of different regions in the enhancer, core promoter, and modulatory regions that removed or added copies of TFBS (Leonard et al., 1989; Michael et al., 1994; Montano et al., 1997; Naghavi et al., 1999). However, such sequence modifications have been sporadic, typically limited to the individual or a small number of viral strains, and cannot be generalized.

One notable exception to this observation is the occurrence of the MFNLP, which primarily represents the duplication of the RBEIII motif in the viral modulatory region with the concomitant co-duplication of the flanking sequences for the binding of other host factors. The RBEIII motif duplication in HIV-1B was found in approximately 38% of primary viral isolates (Estable et al., 1996). Importantly, RBEIII motif duplication in HIV-1B is believed to ensure the presence of a binding site for RBF-2 when the original copy becomes non-functional due to mutations (Chen et al., 2005; Sadowski and Mitchell, 2005).

RBEIII motif duplication in HIV-1C differs from that of HIV-1B in two crucial qualities. First, the creation of an additional RBEIII motif is not associated with the inactivation of the original motif. In other words, nearly all the double-RBEIII viral strains in our cohort of HIV-1C contained two copies of the intact motif without any mutations in the core sequence. Preliminary leads from our laboratory confirm the functional activity of both the motifs in such LTRs. Of note, the participants of the present study are all reportedly ART-naïve by self-declaration. Our data, however, do not rule out the possibility of ART exposure in HIV-1C leading to the inactivation of the original RBEIII motif, necessitating the need for the creation of a second and functional RBEIII motif in the promoter. Second, the co-duplication of the RBEIII and NF-κB motifs is unique to HIV-1C, a property not seen in any other HIV-1 genetic subtype. Thus, HIV-1C appears to exploit the strategy of sequence motif duplication differently compared to other viral subtypes.

Importantly, the addition of more copies of NF-κB to the viral promoter may be beneficial by enhancing the transcriptional strength of the LTR. However, a stronger LTR can be detrimental to maintaining stable latency. HIV-1C appears to have found two different solutions to the paradox of gene expression regulation – limiting the copy number of the NF-κB motifs to three and duplicating the RBEIII motif.

### Limiting the Number of NF-κB Motifs in the Viral Enhancer

Three viral strains, LR-HHC, LR-FHHC, and LR-FHC, lack RBEIII duplication. The prevalence of the LR-FHHC viral strains was only 2% (13 of 607 primary viral isolates) in a southern Indian cohort when discovered during 2000–2003 for the first time (Bachu et al., 2012a). The prevalence of these strains increased to approximately 25% (39/159) during 2010–2011, evaluated at four different clinical sites of India, suggesting transmission success of 4-κB viral strains at the population level (Bachu et al., 2012a). However, in the present study, the prevalence of the LR-FHHC viral strains dropped to 6.2% (28 of 455) during 2017–2019. Notably, a new variant viral group, LR-FHC representing the second-largest proportion among the emerging variants with 8.8% (40 of 455), was identified here for the first time. Given the reduction in the prevalence of LR-FHHC strains and the concomitant appearance of the LR-FHC strains, the former may have originated from the latter.

This observation leads to three logical conclusions. First, LR-FHHC strains, given the stronger transactivation properties of the promoter, may lack replication competence over a sustained period explaining the transient nature of their prevalence in the population. Second, the 4-κB viral strains must relinquish one κB-motif to regain the 3-κB formulation of the enhancer to down modulate the transcriptional strength of the viral promoter. The LR-FHHC strains relinquished one of the two H-κB sites to this end to become LR-FHC. Three, both the canonical LR-HHC and the variant LR-FHC strains contain the same number of NF-κB motifs in the enhancer. However, all the three κB motifs of the FHC-LTR are genetically variable. We propose that the LR-FHC-LTR is likely to be responsive to a broader range of cellular activation signals compared to the LR-HHC-LTR, given the NF-κB motif variation. Thus, by deleting one H-κB site from the LR-FHHC-LTR, HIV-1C appears to have down-modulated transcriptional strength of the viral promoter on the one hand but retained the broader reception potential to cellular signals on the other hand. If the LR-FHC viral strains enjoy a replication advantage at the population level, they are expected to replace the canonical HHC strains in the coming years.

### Is the RBEIII Motif Duplication Essential to Impose Avid Latency of a Stronger Viral Promoter?

Seven different variant LTRs identified in this work contain a second copy of the RBEIII motif added by sequence motif duplication (Figure 1; Supplementary Figure S1D–J). Unlike in HIV-1B where a new RBEIII site is created as a compensatory mechanism when the original copy is mutated (Estable et al., 1996), in HIV-1C, both the RBEIII motifs are, in contrast, intact without a mutation in the core motif (5′-ACTGCTGA-3′). Thus, RBEIII duplication in HIV-1C appears to confer a novel function or an enhanced phenotype of the existing function but not compensating for a loss of function.

A second quality of the RBEIII motif duplication in HIV-1C is also relevant, especially for HIV latency. While RBEIII motif duplication is common to all the HIV-1 genetic subtypes (Figure 2B; Ait-Khaled et al., 1995; Zhang et al., 1997; Estable et al., 1998; Chen et al., 2005), one significant distinction unique to HIV-1C is the co-duplication of the NF-κB motif, not seen in other subtypes. One variant LTR, LRhR-HHC, contains a total of four NF-κB motifs like the old FHHC-LTR. However, the variant LTR contains two RBEIII sites, unlike the LR-FHHC-LTR that has only one (Figure 1). Additionally, the duplicated κB-motif (h-κB) of LRhR-HHC is genetically distinct (5′-GGGACTTTCA-3′) from the other three types (C-, H-, and F-κB) described above. The Single Nucleotide Mutation Model predicted the 5′-GGGACTTTCA-3′ motif to bind the p50 homodimer with reduced affinity compared to the consensus NF-κB motif 5′-GGGACTTTCC-3′. This binding prediction was supported by the bimolecular dsDNA microarray analysis, as demonstrated (Du et al., 2014). Lastly, the duplicated κB-motif of LRhR-HHC-LTR is separated from the viral enhancer by one RBEIII motif thus, obliterating the distinction between the viral modulatory and enhancer elements. The biological significance of creating one of each RBEIII and NF-κB motifs in the LRhR-HHC-LTR is of interest.

In the absence of cell activation, the RBEIII motif functions predominantly as a repressive element by recruiting RBF-2 comprising three different cell factors, including TFII-I (Chen et al., 2005). While TFII-I can activate several cellular genes, it can also suppress gene expression from several other cellular promoters, including c-fos (Roy, 2012). Thus, the presence of two copies of the RBEIII motif in the LTR may have a profound impact on viral latency, probably by stabilizing the latency phase. The NF-κB binding motifs, in contrast, play a predominantly positive role in enhancing transcription from the LTR, under the conditions of cell activation. Thus, a higher copy number of the NF-κB motif (4 copies Vs. 3) in the promoter may offset the negative impact of the RBEIII motifs, especially when the provirus is induced out of latency. Of note, unlike the variant LRhR-HHC, a different variant strain LRhR-HC contains one less NF-κB motif (two RBEIII but only three NF-κB motifs). Preliminary results from our laboratory show that the LRhR-HC-LTR requires a profoundly stronger activation signal, compared to LRhR-HHC-LTR or the canonical LR-HHC-LTR, for latency reversal in Jurkat cells or primary CD4 cells (Bhange et al., unpublished observations).

A different variant promoter pair (LRLR-HHC and LRLR-HC) is also of interest in this respect. This pair also contains an RBEIII motif duplication where the motifs are accompanied by the co-duplication of the TCF-1α/LEF-1 motif, not the NF-κB site. One member of the pair contains three NF-κB motifs (LRLR-HHC), while the other only two (LRLR-HC). This variant promoter pair may have similar gene expression properties as that of the LRhR-HHC- and LRhR-HC-LTRs if the additional copy of TCF-1α/LEF-1 is a functional equivalent of the NF-κB motif.

### The Implication of Promoter Variations for HIV-1 Pathogenesis and Evolution

Since a single promoter regulates the expression of all the HIV-1 proteins and regulates latency, a profound variation in the TFBS composition is expected to have a significant impact on the various properties of the virus, including latency, viral load, disease progression, and viral evolution. The evolution of regulatory elements may play a role as essential or even more important than that of coding sequences (Carroll, 2005). However, little attention has been focused on the evolution of the regulatory elements in HIV-1, than that of the protein-coding regions (Maljkovic Berry et al., 2007).

In our study, the cross-sectional and longitudinal analyses did not find a statistically significant difference in the levels of any of the prognostic markers among the promoter variant strains categorized into four groups, HHC, FHC, FHHC, and RR (Figure 4). A significant difference in PVL and CD4 cell count was found in a previous study from our laboratory when a cohort of 80 patients was divided into HHC and FHHC groups (Bachu et al., 2012a). The present study did not find such differences, except for the RR group manifesting a trend in the complete-case analysis, which was not statistically significant. The analytical power of the present study was profoundly compromised given the loss of study participants due to the implementation of the test and treat policy.

Commensurate with our findings, previous studies of the RBEIII motif duplication in HIV-1B also failed to find an association between the LTR profile and clinical or transcriptional phenotypes in a cross-sectional cohort (Estable et al., 1996). A different study failed to identify a correlation between the RBEIII motif duplication and the syncytium-inducing property of envelope and, thus, disease progression (Koken et al., 1994). Likewise, Koken et al. (1994) reported the lack of an association between the copy number of the RBEIII motif and disease progression.

The coexistence of viral strains could be a likely explanation for the absence of association between the LTR variant forms and prognostic markers in our cohort. Deep sequencing of the samples identified the presence of a coinfection in all four subjects in our study. At present, it appears that the double-RBEIII viral infections are found only as coinfections along with the single-RBEIII strains. In three of the four subjects, single-RBEIII viral strains seem to dominate the double-RBEIII variants in both the genomic DNA and RNA compartments and at most of the follow-up time points. If RBEIII cluster duplication indeed manifests a suppressive effect on viral gene expression, a distinct association between the duplication and the prognostic markers may become evident in a mono-infection, but not in a coinfection. The dominant influence of the RBEIII motif duplication on viral gene expression has been confirmed using panels of engineered viral clones (Bhange et al., unpublished observations).

In summary, our work records the emergence of several promoter variant viral strains in HIV-1C of India over recent years. Sequences representing the variant viral forms are also found in the sequence databases derived from different global regions where HIV-1C is predominant. Sequence motif duplication creates additional copies of TFBS that play a crucial role in regulating HIV latency and even blurs the distinction between the viral enhancer and modulatory regions. Given that the RBEIII and AP-1 sites play a crucial role in regulating latency (Duverger et al., 2013), the influence of the RBEIII motif duplication, especially when accompanied by the co-duplication of the NF-κB motifs, needs experimental evaluation. Consistent monitoring will be necessary to understand which variant viral strains will survive to establish spreading epidemics in the coming years. Detailed investigations are warranted to evaluate the impact of the TFBS profile differences on HIV-1 latency and latent reservoir properties. ART administration may have a profound impact on the promoter variations described here by exacerbating the frequency of such sequence duplications. Further, the present study could not examine the influence of the duration of the viral infection and disease state on promoter variations, as such details are not available from clinics. The present study also restricted the scope of sequence duplication evaluation to the LTR and did not examine sequence motif duplication in the other regions of the viral genome.

## Data Availability Statement

The data sets presented in this study can be found in online repositories. The names of the repository/repositories and accession number(s) can be found in the article.

## Ethics Statement

The studies involving human participants were reviewed and approved by Institutional Ethics Committee of JNCASR, AIIMS, NARI, St. John’s Hospital, and YRG CARE. The patients/participants provided their written informed consent to participate in this study.

## Author Contributions

DB performed research, analyzed data, and wrote the paper. NP, SPM, BPG, SN, DC, BJ, TRD, SFA, NS, AB, and KM performed research. SSi and YG analyzed data. HP analyzed NGS data. PB, BKD, MD, RG, SM, RSP, SSa, AS, SSo, and MT designed research. UR designed research and wrote the paper. All authors contributed to the article and approved the submitted version.

## Funding

This work was supported by the Department of Biotechnology, Ministry of Science and Technology, Government of India (Sanction order no. BT/PR7359/MED/29/651/2012).

## Conflict of Interest

The authors declare that the research was conducted in the absence of any commercial or financial relationships that could be construed as a potential conflict of interest.

## Publisher’s Note

All claims expressed in this article are solely those of the authors and do not necessarily represent those of their affiliated organizations, or those of the publisher, the editors and the reviewers. Any product that may be evaluated in this article, or claim that may be made by its manufacturer, is not guaranteed or endorsed by the publisher.

## Abbreviations

TFBS: Transcription factor binding site
HIV-1C: HIV-1 subtype C
LTR: Long terminal repeats
C-LTR: HIV-1C LTR
NGS: Next-generation sequencing
RT-PCR: Reverse transcription polymerase chain reaction
ART: Antiretroviral therapy
