# Supplementary material for: The Evolution of Regulatory Elements in the Emerging Promoter-Variant Strains of HIV-1 Subtype C
Source: Front Microbiol. 2021 Nov 16;12:779472. doi: 10.3389/fmicb.2021.779472 (PMC8660095; doi:10.3389/fmicb.2021.779472)
Supplement: Supplementary file 1 [file Data_Sheet_1.pdf]

Supplementary information

The evolution of regulatory elements in the emerging promoter-variant strains of HIV-1 subtype C

Disha Bhange[1], Nityanand Prasad[1], Swati Singh[1], Harshit Kumar Prajapati[1], Shesh Prakash Maurya[2], Bindu Parachalil Gopalan[3], Sowmya Nadig[3], Devidas Chaturbhuj[4], Jayaseelan Boobalan[5], Thongadi Ramesh Dinesha[5], Syed Fazil Ahamed[3], Navneet Singh[1], Anangi Brahmaiah[1], Kavita Mehta[1], Yuvrajsinh Gohil[1], Pachamuthu Balakrishnan[6], Bimal Kumar Das[2], Mary Dias[3], Raman Gangakhedkar[7], Sanjay Mehendale[8], Ramesh Paranjape[7], Shanmugam Saravanan[5], Anita Shet[3], Sunil Suhas Solomon[9,10], Madhuri Thakar[4], and Udaykumar Ranga[1]*

[1]HIV-AIDS Laboratory, Molecular Biology and Genetics Unit, Jawaharlal Nehru Centre for Advanced Scientific Research (JNCASR), Bangalore, India

[2]HIV Immunology Laboratory, Department of Microbiology, All India Institute of Medical Sciences (AIIMS), New Delhi, India

[3]Division of Microbiology/ Infectious Diseases Unit, St. John's National Academy of Health Sciences, Bangalore, India

[4]Department of Serology and Immunology, National AIDS Research Institute (NARI), Pune, India

[5]Department of Molecular Biology and Genotyping, Y. R. Gaitonde Centre for AIDS Research and Education (YRG CARE), Chennai, India

[6]Infectious Diseases Laboratory, Y. R. Gaitonde Centre for AIDS Research and Education (YRG CARE), Chennai, India

[7]Department of Clinical Sciences, National AIDS Research Institute (NARI), Pune, India

[8]P. G. Hinduja National Hospital and Medical Research Centre, Mumbai, India

[9]Y. R. Gaitonde Center for AIDS Research and Education (YRG CARE), Chennai, India

[10]Department of Medicine, Johns Hopkins University, School of Medicine, Baltimore, Maryland, United States of America.

***Correspondence:**
Udaykumar Ranga
udaykumar@jncasr.ac.in

Keywords: HIV-1, subtype C, evolution, sequence duplication, latency

Figures

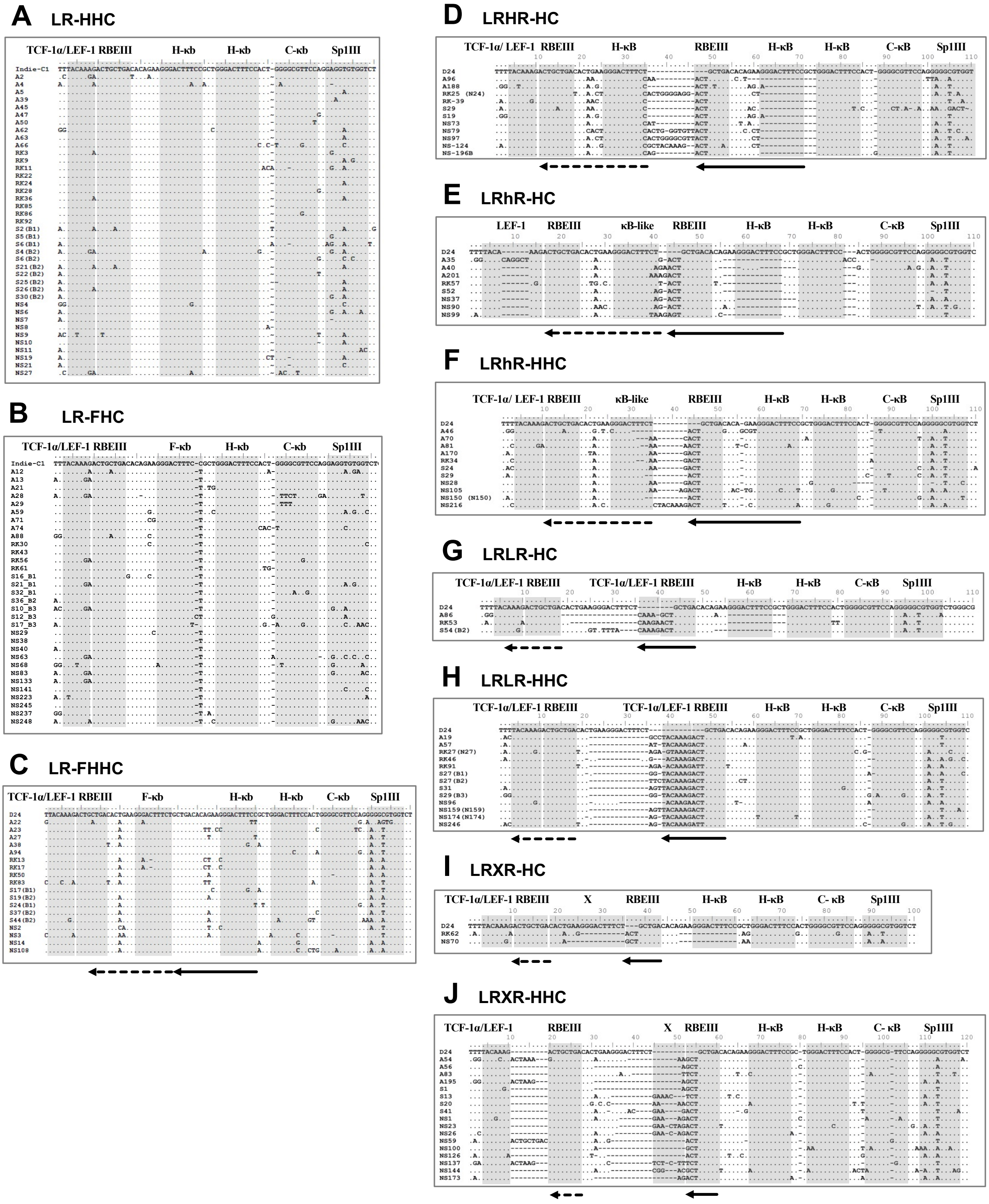

Figure S1: Multiple sequence alignment of the LTR sequences of HIV-1C promoter-variant strains. The alignment contains a few representative sequences under each category. Patient identity is depicted on the left side of the alignment. TFBS of significance are highlighted using grey shade boxes and labeled on the top. **(A)** The LR-HHC (the canonical 3-κB strains) sequences are aligned with the Indie.C1 (AB023804.1) reference sequence. **(B)** The alignment of the LR-FHC variant viral sequences **(C)** The alignment of the LR-FHHC (4-κB variants) sequences with the D24 (EF469243.2 reference sequence. **(D, E, F, G, H, I and J)** Sequence alignment of seven types of double-RBEIII variant viral strains. The intervening sequences between the two RBEIII sites represent an H-κB (H), κB-like (h), hLEF (L), or un-typable (X) motif. The solid and dotted arrows represent an original and duplicated sequence, respectively.

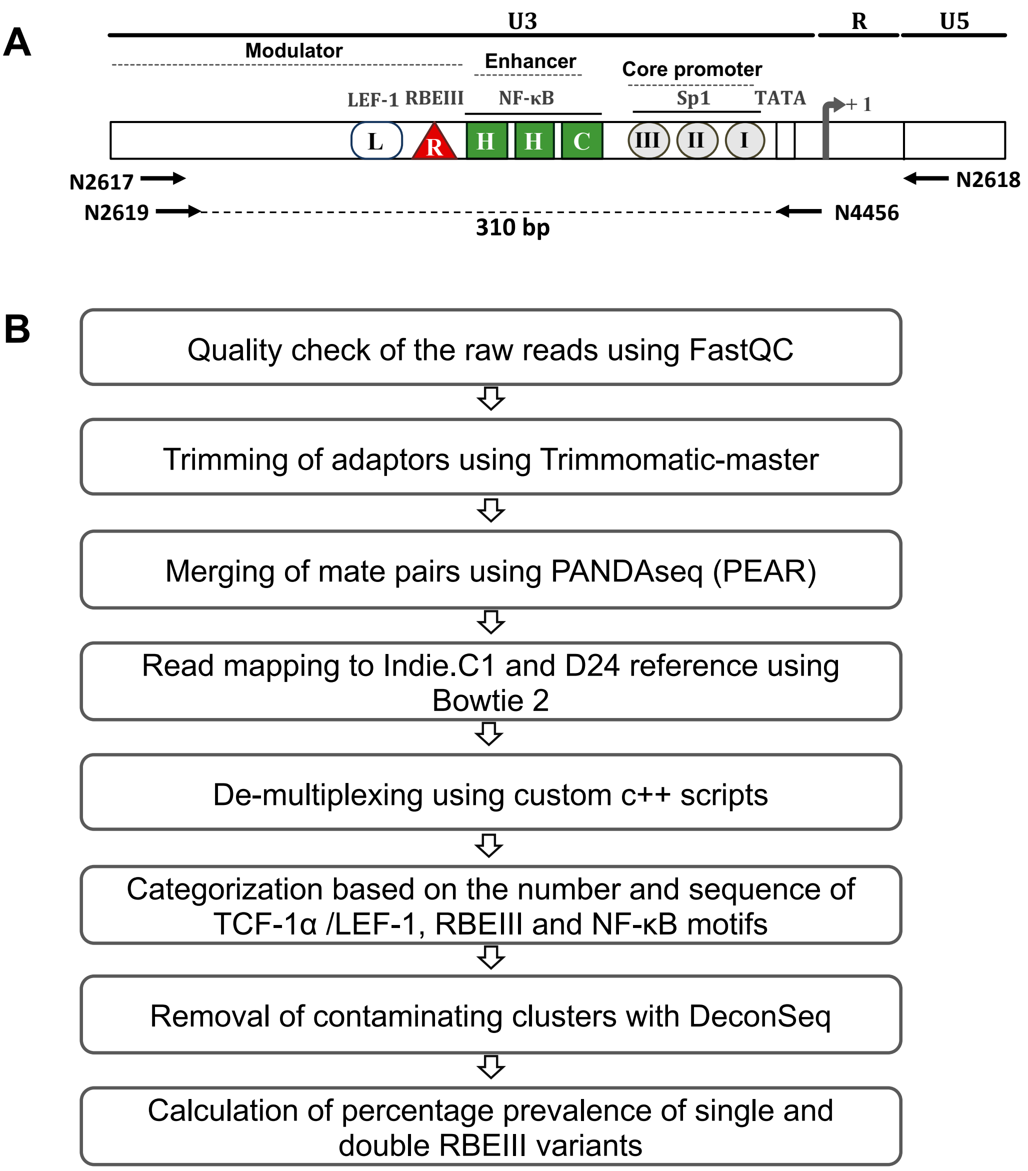

Figure S2: A schematic representation of the PCR amplification and Illumina sequencing of the LTR. **(A)** The location and orientation of the primers amplifying the LTR region are depicted. A PCR fragment of 310 bp was used for the NGS. **(B)** The PCR fragments were analyzed using the pair-end MiSeq Illumina sequencing. The flowchart depicts the pipeline followed for data analysis. The reads were mapped to the reference sequence of Indie.C1.

Tables

Table S1. Primers used for PCR and cDNA synthesis

Purpose		Primer no.	Sequence (5′-3′)
**Primary screening**	OFP	N2617M	CCACTGACCTTTGGATGGTGCTTCAAG
	ORP	N2618	TGAGGGATCTCTAGTTACCAGAGTCAC
	IFP	N2619	TGCTTCAAGCTAGTRCCAGTTGACCCA
	IRP	N2620	AGAGTCACACAACAGACGGGCACACA
**Sanger sequencing**		N2621	AAGCTGGTACCAGTTGAC
**cDNA synthesis**		N4101	TTTTTTTTTTTTTTTCAGAGCACTC
		N4102	TTTTTTTTTTTTTCAGAGCACTCAAG
		N4103	TTTTTTTTTTTTTTCAGAGCACTCAAGG
**NGS**	OFP	N2619	TGCTTCAAGCTAGTRCCAGTTGACCCA
	ORP	N2618	TGAGGGATCTCTAGTTACCAGAGTCAC
	IFP	N2619BX	(8 bp barcode)-TGCTTCAAGCTAGTRCCAGTTGACCCA
	IRP	N4456BX	(8 bp barcode)- GCGAAAAGCAGCTGCTTATATGCAGCATCTGAGGG

OFP= Outer forward primer, ORP= Outer reverse primer, IFP= Inner forward primer, IRP= Inner reverse primer, X= Number of the barcode

Table S2: (A) Combination of barcoded primers used for LTR amplification from each sample **(B)** Sequence of each 8 bp barcode

A

Sample No.	Month	Genomic DNA		Plasma RNA	
		Set 1	Set 2	Set 1	Set 2
**2079**	M0	B5 F	B6 F	B5 F	B6 F
		B1 R	B1 R	B12 R	B12 R
	M6	B5 F	B6 F	B5 F	B6 F
		B4 R	B4 R	B13 R	B13 R
	M12	B5 F	B6 F	B5 F	B6 F
		B7 R	B7 R	B15 R	B15 R
**4084**	M0	B1 F	B2 F	B1 F	B2 F
		B4 R	B4 R	B7 R	B7 R
	M6	B1 F	B2 F	B1 F	B2 F
		B5 R	B5 R	B11 R	B11 R
	M12	B1 F	B2 F	B1 F	B2 F
		B6 R	B6 R	B12 R	B12 R
**3767**	M0	B3 F	B4 F	B3 F	B4 F
		B5 R	B5 R	B11 R	B11 R
	M6	B3 F	B4 F	B3 F	B4 F
		B6 R	B6 R	B12 R	B12 R
	M12	B3 F	B4 F	B3 F	B4 F
		B7 R	B7 R	B13 R	B13 R
**VFSJ20**	M0	B13 F	B14 F	B13 F	B14 F
		B19 R	B19 R	B1 R	B1 R
	M12	B13 F	B14 F	B13 F	B14 F
		B20 R	B20 R	B4 R	B4 R
	M24	B13 F	B14 F	B13 F	B14 F
		B22 R	B22 R	B7 R	B7 R

B

Barcode No.	Sequence (5'-3')
B1	TCGCCTTA
B2	CTAGTACG
B3	TTCTGCCT
B4	GCTCAGGA
B5	AGGAGTCC
B6	CATGCCTA
B7	GTAGAGAG
B9	TGCCTCTT
B10	TCCTCTAC
B11	TCATGAGC
B12	CCTGAGAT
B13	TAGCGAGT
B14	GTAGCTCC
B15	TACTACGC
B19	GAGCGCTA
B20	CGCTCAGT
B22	ACTGATCG
